# Research and Regulatory Issues for Integrative Oncology

**DOI:** 10.3747/co.v15i0.273

**Published:** 2008-08

**Authors:** S.M. Sagar, R.K.W. Wong

**Affiliations:** McMaster University and the Juravinski Cancer Centre, Hamilton, Ontario, Canada

**Keywords:** Integrative oncology, cam, research, regulation, acupuncture, natural health products, herbs

## Abstract

Many oncology patients are empowering themselves to self-treat with herbs, nutritional supplements, and mind–body techniques. Other practitioners, such as acupuncturists, are becoming involved in the supportive care of cancer patients. Government research agencies are supporting studies that evaluate complementary therapies. This educational article provides an overview of the challenges in designing appropriate studies of complementary and alternative therapies, evaluating the results, and regulating implementation of useful therapies.

